# Heatwave‐induced synchrony within forage fish portfolio disrupts energy flow to top pelagic predators

**DOI:** 10.1111/gcb.15556

**Published:** 2021-03-06

**Authors:** Mayumi L. Arimitsu, John F. Piatt, Scott Hatch, Robert M. Suryan, Sonia Batten, Mary Anne Bishop, Rob W. Campbell, Heather Coletti, Dan Cushing, Kristen Gorman, Russell R. Hopcroft, Kathy J. Kuletz, Caitlin Marsteller, Caitlin McKinstry, David McGowan, John Moran, Scott Pegau, Anne Schaefer, Sarah Schoen, Jan Straley, Vanessa R. von Biela

**Affiliations:** ^1^ U.S. Geological Survey Alaska Science Center Juneau AK USA; ^2^ U.S. Geological Survey Alaska Science Center Anchorage AK USA; ^3^ Institute for Seabird Research and Conservation Anchorage AK USA; ^4^ NOAA Alaska Fisheries Science Center Auke Bay Lab Juneau AK USA; ^5^ Marine Biological Association Nanaimo BC Canada; ^6^ Prince William Sound Science Center Cordova AK USA; ^7^ Southwest Alaska Inventory and Monitoring Network National Park Service Fairbanks AK USA; ^8^ Pole Star Ecological Research LLC Anchorage AK USA; ^9^ College of Fisheries and Ocean Sciences University of Alaska Fairbanks Fairbanks AK USA; ^10^ Migratory Bird Management U.S. Fish and Wildlife Service Anchorage AK USA; ^11^ NOAA Alaska Fisheries Science Center Seattle WA USA; ^12^ University of Alaska Southeast Sitka AK USA

**Keywords:** ecosystem response, forage fish, Gulf of Alaska, marine heatwave, portfolio effects

## Abstract

During the Pacific marine heatwave of 2014–2016, abundance and quality of several key forage fish species in the Gulf of Alaska were simultaneously reduced throughout the system. Capelin (*Mallotus catervarius*), sand lance (*Ammodytes personatus*), and herring (*Clupea pallasii*) populations were at historically low levels, and within this community abrupt declines in portfolio effects identify trophic instability at the onset of the heatwave. Although compensatory changes in age structure, size, growth or energy content of forage fish were observed to varying degrees among all these forage fish, none were able to fully mitigate adverse impacts of the heatwave, which likely included both top‐down and bottom‐up forcing. Notably, changes to the demographic structure of forage fish suggested size‐selective removals typical of top‐down regulation. At the same time, changes in zooplankton communities may have driven bottom‐up regulation as copepod community structure shifted toward smaller, warm water species, and euphausiid biomass was reduced owing to the loss of cold‐water species. Mediated by these impacts on the forage fish community, an unprecedented disruption of the normal pelagic food web was signaled by higher trophic level disruptions during 2015–2016, when seabirds, marine mammals, and groundfish experienced shifts in distribution, mass mortalities, and reproductive failures. Unlike decadal‐scale variability underlying ecosystem regime shifts, the heatwave appeared to temporarily overwhelm the ability of the forage fish community to buffer against changes imposed by warm water anomalies, thereby eliminating any ecological advantages that may have accrued from having a suite of coexisting forage species with differing life‐history compensations.

## Graphical abstract

The Pacific marine heatwave was a prolonged warming event that led to reduced abundance and quality of small pelagic forage fishes. This caused a major disruption in the pelagic marine food web that precipitated large‐scale mortality and reproductive failures in upper trophic level predators. Unlike decadal‐scale variability underlying ecosystem regime shifts, the heatwave appeared to temporarily overwhelm the ability of the middle trophic level to buffer against changes imposed by warm water anomalies.

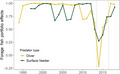

## 1 INTRODUCTION

The Pacific marine heatwave (MHW) of 2014–2016 was among the largest and most persistent MHWs documented globally to date. It was characterized by ocean temperatures >2 SD above normal over an area spanning two large marine ecosystems and sustained across multiple winters (Bond et al., 2015; Di Lorenzo & Mantua, 2016; Hobday et al., 2018). The MHW had formed in the upper 100 m of the water column over the central Gulf of Alaska (GOA) by January 2014 (Freeland & Whitney, 2014), had reached coastal regions by the summer, and was largely confined to the inner shelf by September that year (Walsh et al., 2018). The warm water anomaly had deepened across the water column by early 2015 and was enhanced during the winter of 2015–2016 by one of the strongest El Niño events on record (Joh & Di Lorenzo, 2017).

The extreme MHW impacted marine organisms at multiple trophic levels (Barbeaux et al., 2020; Brodeur et al., 2019; Daly et al., 2017; Peterson et al., 2017; Santora et al., 2020), and a growing body of evidence indicates an abrupt ecosystem‐wide response occurred over the large spatial extent (Suryan et al., 2021; Walsh et al., 2018). Large‐scale mortality events by marine predators and pelagic food web specialists were among the first visible indicators of a major ecosystem perturbation in the GOA during the MHW (Piatt et al., 2020; Savage, 2017) and suggested a major disruption in energy transfer through the middle trophic level.

Forage fish, which play a key intermediary role in energy transfer from plankton to predators, are small pelagic species generally known for “boom and bust” population cycles in which repeated collapse and recovery can occur over long time periods (McClatchie et al., 2017). Similar to a collection of assets in a financial portfolio (Figge, 2004; Schindler et al., 2010), diversity within forage fish communities reduces the variance of the ecosystem services they provide to pelagic marine food webs. Asynchrony within the forage fish portfolio tends to dampen the effects of large fluctuations in abundance by any one species at a time. Niche differentiation, which can arise from decreased interspecific competition or decreased correlations among species‐specific responses to environmental conditions, tends to increase asynchrony in population trajectories among species and leads to greater stability within communities (Loreau & De Mazancourt, 2008). Portfolio effects, which provide a measure of the covariance among species within a community, can be used as a framework for understanding stability in ecosystem dynamics over time (Schindler et al., 2015; Thorson et al., 2018).

**FIGURE 1:**
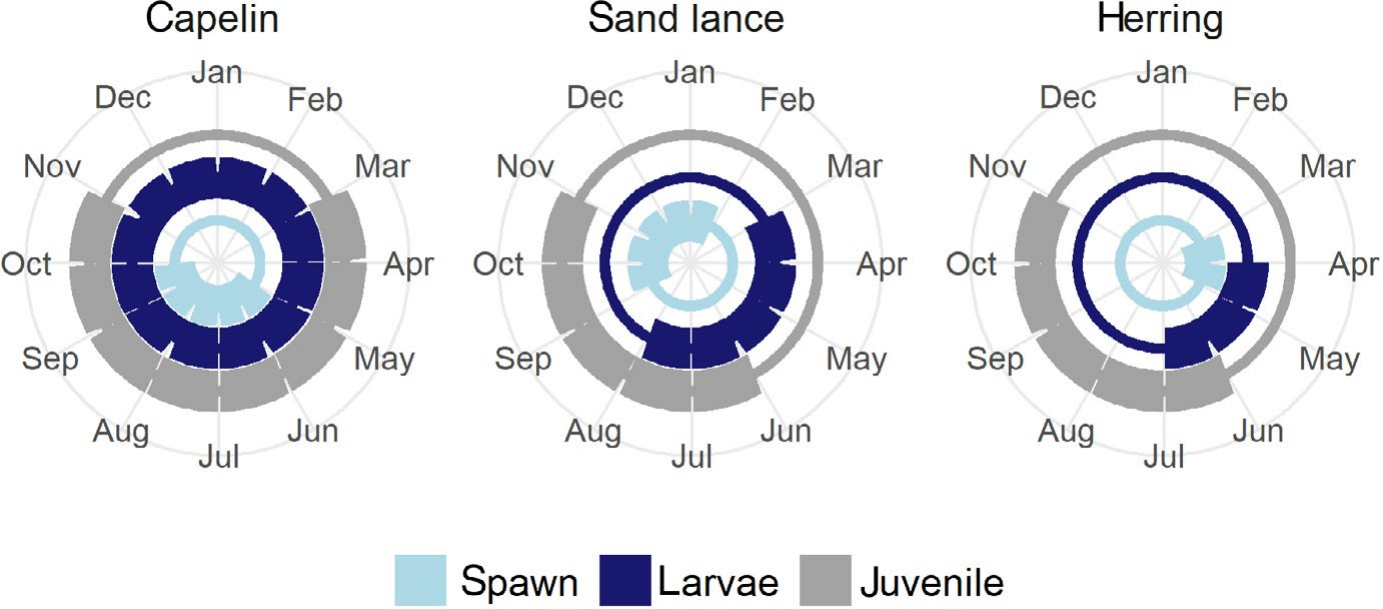
Generalized life‐history strategies, including monthly timing of spawning, larvae, and early juvenile stages, for three key forage fish species in the North Pacific

Major disruptions in energy transfer through forage fish communities are rare because diversity of species with different life‐history strategies promotes resilience in marine food webs (Kondoh, 2003; Perry et al., 2010). For example, variability among forage species in migration, age at maturity and seasonality of life stages (Figure 1), and timing of energy allocation toward growth and storage (Heintz et al., 2013; Norcross et al., 2001) can help to mitigate forage fish predators against bottlenecks at middle trophic levels.

Life histories of the three main GOA forage species, Pacific capelin (*Mallotus catervarius*), Pacific herring (*Clupea pallasii*), and Pacific sand lance (*Ammodytes personatus*), hereafter capelin, herring, and sand lance, respectively, are fairly well known. Capelin migrate from the continental shelf to spawn along beaches over protracted periods from spring through fall (Arimitsu et al., 2008; Brown, 2002). Capelin larvae overwinter, a strategy that favors transformation into their juvenile form at a relatively large size, and development during the peak of productivity in spring (Doyle et al., 2019). It also allows for growth and lipid storage in summer/fall periods at age‐1 (Figure 1). Herring is also a migratory species, as juveniles are reared inshore but move offshore prior to returning as adults, primarily at age‐3+, to spawn during spring. Their larval development occurs rapidly during spring, and transformation to their juvenile state occurs by their first summer at age‐0 (Doyle et al., 2019). Growth of juveniles during summer and fall is critical to successful overwintering of herring (Heintz et al., 2013; Sewall et al., 2019). Sand lance is a non‐migratory species associated with nearshore sandy bottom habitats (Baker et al., 2018; Ostrand et al., 2005) that spawns from fall to winter months (Robards, Anthony, et al., 1999; Robards, Piatt, et al., 1999). Hatch timing in sand lance is mediated by temperature, with larvae undergoing rapid growth during spring (Doyle et al., 2019). Like herring, the energy allocation strategy favors juvenile growth of sand lance during the summer and fall prior to their first winter at age‐0, and subsequently switches to lipid storage during age‐1 (Robards, Anthony, et al., 1999; Robards, Piatt, et al., 1999). Although any forage fish life stage can be targeted, adult spawning aggregations tend to concentrate marine predators in space and time (Womble et al., 2005).

In this study, we investigate changes within the forage fish community that manifested in the northern GOA during the MHW, including variation in abundance and changes in age structure, size, growth, and energy content of species common in diets of pelagic predators. Incorporating new data from long‐term monitoring efforts within the region, we put these forage fish observations into context with coincident changes in zooplankton and upper trophic level predators. Finally, with a focus on the pelagic trophic pathway in the GOA, we identify potential mechanisms that underlie mass mortalities and reproductive failures in top predators that were associated with the MHW (Barbeaux et al., 2020; Piatt et al., 2020; Savage, 2017).

## 2 MATERIALS AND METHODS

### 2.1 Study area

**FIGURE 2:**
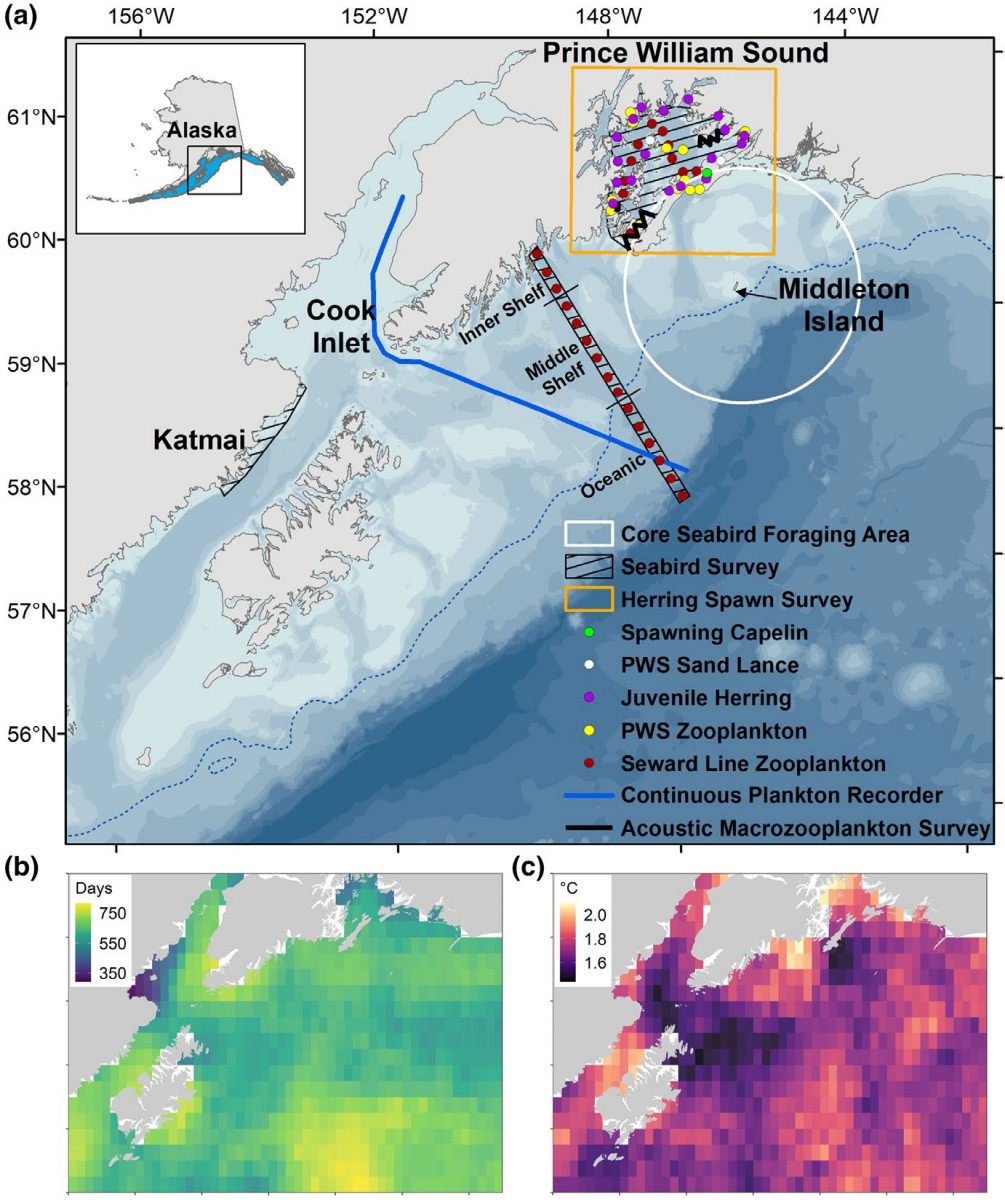
(a) Map of the study area, including sampling locations in Prince William Sound (PWS) and the northern Gulf of Alaska (GOA). Capelin survey extent includes the larger Gulf of Alaska region (blue shaded area, inset). Seafloor depth reaches over 4500 m in the study area (>700 m in PWS) with lighter shading representing shallower depths, the 500 m isobath is denoted by a dashed‐blue line. Distribution of marine heatwave event indices during 2014–2016 including (b) cumulative number of heatwave days, and (c) mean of long‐term (1982–2018) sea surface temperature anomalies for heatwave events in each pixel

The study area includes the northern GOA, Prince William Sound (PWS), Cook Inlet, and the offshore region between 143–156°W and 56–61°N (Figure 2a). Circulation in the coastal and shelf regions of the northern GOA is influenced by the Alaska Coastal Current, the dominant alongshore current, as well as wind mixing and freshwater input (Stabeno et al., 2016; Weingartner et al., 2005). In PWS, with basin depths exceeding 700 m, the Alaska Coastal Current also defines transport from east to west through two ocean entrances in PWS, although similar to conditions on the shelf, circulation is mediated by seasonal and interannual variation in winds and freshwater runoff (Niebauer et al., 1994). Dynamic ocean conditions characteristic of the region are driven in part by complex bottom topography on the shelf and coastal embayments (Figure 2a). Strong winter downwelling relaxes seasonally, which enriches nutrient supply to the shelf and promotes spring and fall bloom activity that are important in driving productivity in this system (Childers et al., 2005; Waite & Mueter, 2013).

### 2.2 MHW indices

Marine heatwave events within in the study area were defined as anomalously warm events lasting five or more days, with sea surface temperatures (SST) warmer than the 90th percentile (Hobday et al., 2016). Daily ¼ degree SST data (NOAA OISST 1982–2018, Table 1; Huang et al., 2020) from the northern GOA region were extracted and analyzed using the R package “heatwaveR” (Schlegel & Smit, 2018). To identify the spatial extent, duration, and magnitude of the MHW, we summarized the total number of heatwave days and the mean intensity (temperature anomaly, °C) of events within each grid cell.

### 2.3 Forage fish abundance indices

#### 2.3.1 Seabird diets

Information on predator diets are among provide some of the longest time‐series data for most forage species in the region (Hatch & Sanger, 1992; Piatt et al., 2018; Sydeman et al., 2017). Seabird diet samples were collected from adults provisioning chicks during the breeding season (June–August) from 1993 to 2018 using methods described previously (Hatch, 2013, 2019; Schoen et al., 2018; Thayer et al., 2008). Frequency of occurrence in diets of obligate surface feeders (black‐legged kittiwakes, *Rissa tridactyla*) and divers (rhinoceros auklets, *Cerorhinca monocerata*, and tufted puffins, *Fratercula cirrhata*) provide indices of availability for preferred prey species (Piatt et al., 2018) within the approximate foraging range that encompasses much of the continental shelf and coastal waters around the colony at Middleton Island (Table 1, Figure 2a; Cunningham et al., 2018; Osborne et al., 2020). For each seabird feeding guild, availability indices (frequency) were calculated by forage fish species and year.

We used portfolio effects as a measure of synchrony among frequencies of these preferred forage fish species for each seabird feeding guild (Barnes et al., 2020; Loreau & de Mazancourt, 2008; Thorson et al., 2018). To identify patterns across relatively short time‐scales, we calculated variance ratios VR_*i*_ *,_j_* for each of *i* 3‐year centered rolling time windows and *j* feeding guilds such that:

$${\text{VR}}_{i,j}=\frac{\text{var}\left(\sum F_{i,j}\right)}{\sum \text{var}\left(F_{i,j}\right)},$$

where $\mathrm{var}\left(\sum F_{i,j}\right)$ is the variance of the frequencies summed across forage fish species at time window *i*, for feeding guild *j*, and $\sum \mathrm{var}\left(F_{i,j}\right)$ is the sum of the variance of frequencies summed across forage fish species at time window *i* for feeding guild *j*. Portfolio effects were calculated as 1 − ${\mathrm{VR}}_{i,j}$ so that lower values indicate greater synchrony across species and therefore lower trophic stability in the system (Thorson et al., 2018).

#### 2.3.2 Survey indices

Most forage fish species in the region have not been commercially exploited in the region owing to conservation measures put in place in the late‐1990s (Witherell et al., 2000), and therefore directed surveys to inform management of these species are limited. The exception is herring in PWS, which had historically been fished for more than a century but has been closed since 1999 due to insufficient biomass (Muradian et al., 2017). Survey‐based indices of abundance were available for capelin in the northern GOA, which are sampled incidentally in fisheries surveys targeting other species, and for PWS herring, however, no survey data exist for sand lance (Table 1).

Variability in capelin relative abundance over the GOA continental shelf was examined using annual estimates of mean densities of age‐1+ capelin from three fisheries‐independent surveys conducted by the National Oceanic and Atmospheric Administration Alaska Fisheries Science Center between 2000 and 2019. Indices of relative abundance based on mean CPUE (kg km^−2^) for age‐1+ capelin (>60 mm fork length) biomass were calculated from the summer GOA walleye pollock (*Gadus chalcogrammus*) acoustic‐trawl survey, the late‐summer small‐mesh midwater trawl survey (hereafter pelagic trawl survey), and the summer GOA bottom trawl survey over the shelf and upper slope (<500 m depth) following McGowan et al. (2020). While these surveys were not designed specifically to sample capelin, they collectively track years of relatively high and low capelin abundance.

Trends in PWS spawning herring stock biomass are inferred from Alaska Department of Fish and Game's annual spring spawning surveys (mile‐days of milt; Haught & Moffit, 2019), which provides fishery independent data for the age‐structured stock assessment model (Muradian et al., 2017).

### 2.4 Forage fish quality indices

#### 2.4.1 Spawning capelin size at age

Age structure of spawning capelin from PWS was assessed from otoliths of fish collected in July (Table 1; Arimitsu et al., 2017). Samples were collected with a dip net in 2013 and a cast net in 2016, both targeting schools under feeding seabirds within 2 km of a known beach spawning site at one of two main entrances connecting the GOA and PWS (Figure 2a). No capelin were observed there in 2014 and 2015. Age was assigned by counting translucent zones in sagittal otoliths. To reduce variability associated with sexual dimorphism in spawning capelin, we identified changes in the distribution of length at age (total length, mm) for male capelin in spawning condition based on external morphology (i.e., raised lateral line and enlarged anal fin).

#### 2.4.2 Sand lance energy content, age, and size structure

We assessed age and energy content of sand lance sampled by nets in PWS 2012–2016 and 2018 (Table 1; Arimitsu et al., 2017) using the methods described by von Biela et al. (2019). Age was assessed from otoliths (Robards et al., 2002), and energy content of age‐1 fish was assessed with bomb calorimetry. Whole‐body total energy (kJ) was estimated by multiplying the energy density (kJ g^−1^ dry mass) by dry mass (g), and then compared among years with ANOVA and Tukey's HSD post hoc test (*α* < 0.05).

Because age data were not available for the entire time series, we used length data (total length, mm) from sand lance sampled by seabirds at Middleton Island to identify changes in size structure over time. Diving auklets and puffins deliver whole and undigested prey samples to their chicks, which allows for measurements of fish length and mass (Thompson et al., 2019). We used lognormal mixture models (MacDonald & Pitcher, 1979) to estimate the proportion of multimodal size classes with annual length frequency data and multiplied proportions for each size class by the CPUE in each year. The first size group contained fish <100 mm in length and typically corresponds to age‐0 fish in summer. Age classes of the second and third size groups could not be determined reliably from length (von Biela et al., 2019), but are assumed to be between age‐1 and age‐4 (Robards, Anthony, et al., 1999; Robards, Piatt, et al., 1999). Analyses were conducted with the R package “mixtools” (Benaglia et al., 2009).

#### 2.4.3 Herring growth and energy content

Scale growth increment measurements were used to document long‐term patterns of growth in herring from 1978 to 2017 (Table 1; Alaska Department of Fish & Game, 2019; Batten et al., 2016). Age, sex, and length data were collected from commercial fisheries (1978–1981) and fishery independent (1982–2017) research projects outlined by Baker et al. (1991). Samples were collected with purse seines and cast nets periodically through the spawning season, targeting a sample size of 450 fish for measurements of standard length (mm), weight (g), and sex. Maturity was determined from the gonads. Scales were mounted on slides, annuli counted to determine age, and archived by the Alaska Department of Fish and Game. A subset of these scales was imaged, with a target of 180 scales (30 each from males and females age‐4 through age‐6) from each year. Scales were photographed or scanned at known magnification, and the width of growth increments were measured digitally with Image‐Pro software, to determine the distance between adjacent annuli. Age and sex were not significantly related to growth (ANOVA, *p* = 0.82 and 0.69, respectively) so we pooled the data for further analysis.

Energetics data from juvenile herring identify seasonal changes in total energy content. Herring samples were collected in nursery areas (Figure 2a) in November and March 2007–2016, and processed for energetics using bomb calorimetry and carbon to nitrogen ratios (Gorman et al., 2016, 2018). For individuals 55–115 mm in fork length, energy density (kJ g^−1^) was calculated as the carbon/nitrogen atomic ratio × 0.103 + 32.6 × dry/wet weight ratio −2.902, and total energy (kJ) was calculated as energy density × net wet weight (g). Due to unbalanced sampling across sampling sites and time periods (Gorman et al., 2018), we applied a linear generalized mixed effects model for each month to estimate log‐transformed total energy by year while accounting for random differences in intercepts among sample locations nested in year. We then estimated least square means of back‐transformed total energy for each year. For these analyses, we used “lme4” and “emmeans” packages in R version 4.0.2 (Bates et al., 2015; Lenth, 2020; R Core Team, 2020).

### 2.5 Zooplankton abundance indices

To identify coincident changes in seasonal zooplankton standing stock during the MHW, we summarized trends in euphausiids and copepod abundance indices from several sources (Table 1). These include multinet samples from the Seward Line sampling effort on the GOA shelf and PWS (Hopcroft et al., 2018) and Continuous Plankton Recorder (CPR) data from along the GOA shelf (Batten, 2019), multinet, or bongo tows from within PWS (Campbell & McKinstry, 2019), and acoustic data (Arimitsu et al., 2017).

#### 2.5.1 Euphausiids

We summarized the mean biomass of euphausiids by species from 2012 to 2017 along the GOA shelf on the Seward Line and associated sampling in PWS (Figure 2a) during spring (May) and fall (September). These samples were collected at night using a 500‐µm mesh Hydrobios multinet system towed from 100 m depth (Sousa et al., 2016). To identify spatial differences along the GOA shelf and in PWS, we summarized the station‐level data by season and region: PWS (stations within the large embayment), and Inner Shelf region (shore to 50 km from shore), which is heavily influenced by the Alaska Coastal Current; the transitional Middle Shelf region (50 km from shore to the shelf‐break, defined using the 1000 m depth contour); and the Oceanic region (slope and basin, beyond the 1000 m depth contour).

#### 2.5.2 Copepods

We summarized patterns in the seasonal abundance of copepods using monthly data from CPR (Batten et al., 2018) samples from 2004 to 2017 and bongo net tows in PWS (McKinstry & Campbell, 2018) from 2009 to 2018 (Figure 2a). Species groupings for CPR data included small (<2 mm adult total length) and large (≥2 mm total length) copepods. For PWS data, we included large copepods *Neocalanus* spp. that dominate in spring, and also defined two subgroups: warm water copepods typically found in the California Current including *Calanus pacificus*, *Clausocalanus* spp., *Corycaeus anglicus*, *Ctenocalanus* spp., *Mesocalanus tenuicornis*, and *Paracalanus parvus* (Batten & Walne, 2011; Fisher et al., 2015; Hooff & Peterson, 2006), and resident species, including *Calanus marshallae*, *Neocalanus cristatus*, *N. flemingeri*, *N. plumchrus,* and *Eucalanus bungii*.

#### 2.5.3 Acoustic macrozooplankton

We also classified macrozooplankton along acoustic transects in PWS during September using multifrequency acoustic methods. Acoustic surveys were conducted using Simrad EK60 (Kongsberg Maritime) echosounder with a towed (2014, 2017–2018) or hull mounted (2019) split‐beam transducers (38 and 120 kHz, 12° and 7° beam widths, respectively, measured at half‐power) along transects in three sub‐regions that typically contain foraging aggregations of humpback whales (*Megaptera novaeangliae*; Figure 2a). The echosounder system was calibrated during each survey by suspending a 38.1‐mm tungsten carbide sphere following standard practices (Foote et al., 1987). Acoustic backscatter in the water column were classified for depths between 4 m below the surface and 1 m above the bottom using frequency response methods for inshore waters (De Robertis & Ormseth, 2018). Briefly, the difference in mean volume backscattering strength (*S*_v_, dB re 1 m^−1^, hereafter dB) between 120 and 38 kHz data (Δ*S*_v120 kHz–38 kHz_) in each 5 ping by 5 m acoustic sample was computed in the log domain using a minimum threshold of −80 dB. Samples with ΔS_v120 kHz–38 kHz_ values between 8 and 30 dB were classified as macrozooplankton based on known acoustic scattering characteristics (De Robertis et al., 2010). For each 5 m deep by 0.5 km horizontal increment along transects (150 km total) in each region and year, we computed the acoustic macrozooplankton index as the mean nautical area scattering coefficient (NASC, m^2^ nmi^−2^) from the 120 kHz data. In addition to data from fixed transects in 2017–2019, we also included data from the 2014 pilot work which overlapped in space but included fewer samples (e.g., in Montague Strait, Figures S1 and S2).

### 2.6 Marine predators

We identified changes in distribution and abundance of two conspicuous marine predators that experienced mass mortalities as one of the first conspicuous signs that an ecosystem disruption was underway during the MHW (Piatt et al., 2020; Savage, 2017). Humpback whales and murres (*Uria* spp., seabirds) are pelagic food web specialists that feed on seasonally available forage fish and euphausiids (Hobson et al., 1994; Sanger, 1987; Straley et al., 2018).

#### 2.6.1 Humpback whale

Interannual variability in humpback whale encounter rates (individuals km^−1^ surveyed) were determined from fall (September) surveys in PWS from 2007–2019 (Table 1; Moran et al., 2018; Moran & Straley, 2019). In addition, the number of humpback calves observed in relation to adult counts provides an index of reproductive rates over time.

#### 2.6.2 Common murre

To identify recent changes in the distribution of murres, at‐sea surveys were conducted during 2006–2019 using strip transect methods in nearshore areas of Katmai National Park and Preserve in late‐winter (March) and summer (June–July; Coletti & Kloecker, 2017), and along the Seward Line GOA shelf and in PWS in spring (May), and early fall (September), and within PWS during late fall (October–November) and late winter (February–March; Table 1; Bishop & Shaeffer, 2019; Stocking et al., 2018). Transects were split into ~3‐km segments to calculate densities (birds km^−2^), averaged, and standardized to a mean of 0 and SD of 1 by season and year for each of the following regions: Katmai, PWS, Inner Shelf, Middle Shelf, and Oceanic.

## 3 RESULTS

### 3.1 MHW duration, magnitude, and extent

During 2014–2016, MHW events summarized within each ¼ degree resolution grid cell in the northern GOA ranged in cumulative duration from 291 to 813 days (mean ± SD: 646 ± 67 days). The mean intensity across all events within a given grid cell (n = 788) ranged from 1.4 to 2.1°C (1.7 ± 0.1°C). The distribution of MHW events varied in duration and intensity over the 2 years (Figure 2b,c), with longest cumulative heatwave days observed in offshore regions. Greatest heatwave intensity occurred in some coastal areas, such as northwestern PWS, which is typically influenced by glacial runoff, and Shelikof Strait adjacent to the Katmai Coast. Areas with lower average intensity heatwaves included Lower Cook Inlet and the region south of the western entrance to PWS.

### 3.2 Forage fish availability in seabird diets

Across all years, 79% (*n* = 5281) of diver diet samples and 76% (*n* = 3936) of surface feeder diet samples contained at least one capelin, sand lance, or herring, establishing these species as the dominant seabird prey species at Middleton Island. Correlations between diets of divers and surface feeders differed by species: capelin (*r* = 0.84, *p* < 0.001) and sand lance (*r* = 0.85, *p* < 0.001) than for herring (*r* = 0.36, *p* = 0.088).

**FIGURE 3:**
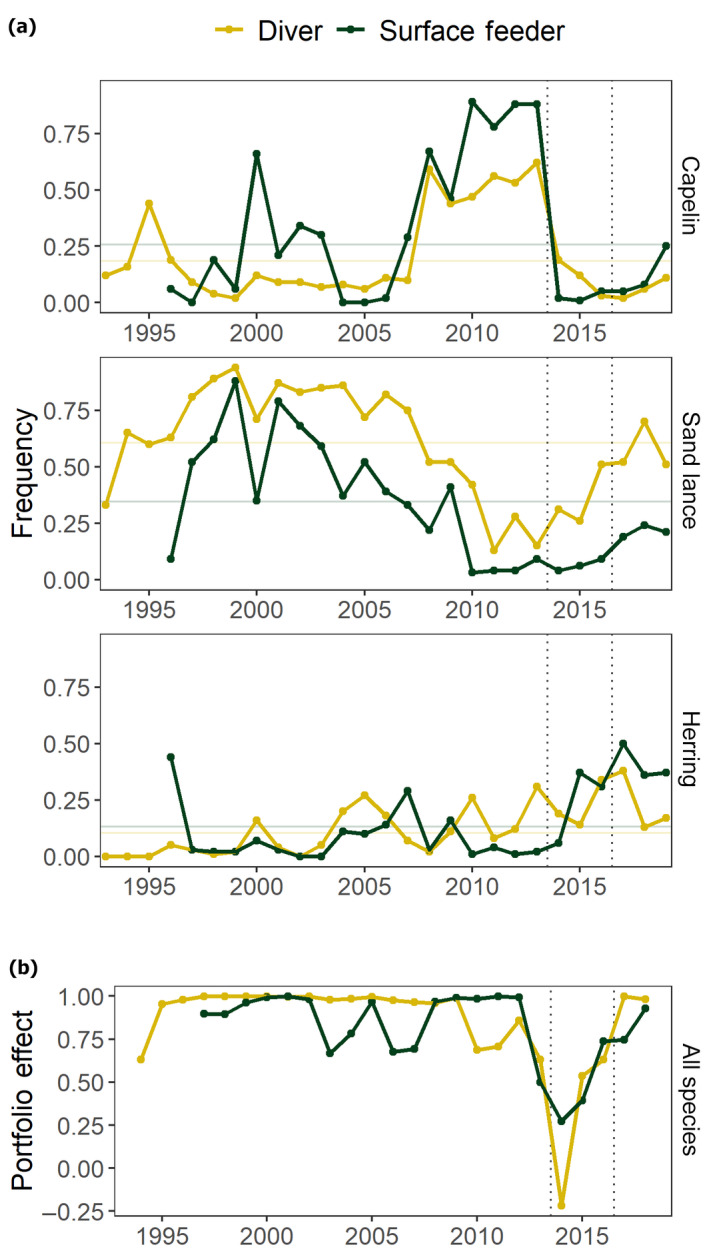
(a) Forage fish availability indices (frequency of occurrence) in seabird diets by feeding guild (color) at Middleton Island. Mean values across all years are shown as horizontal lines, and dotted vertical lines demarcate the timing of the Pacific Marine Heatwave. (b) Portfolio effects as a measure of the degree of synchrony among capelin, herring, and sand lance indices by seabird feeding guild. Portfolio effects were computed from variance ratios across 3‐year centered rolling windows, with lower values indicating greater synchrony and lower buffering capacity by the forage fish community

The frequency of capelin in seabird diets was increasing over several years (2008–2013) before the MHW, abruptly declined by 2014, and remained low through 2018. Decline in the frequencies of capelin between 2013 and 2014 was more abrupt in obligate surface feeding seabirds (from 0.88 to 0.02) than in diving seabirds (from 0.62 to 0.19; Figure 3a), indicating that at least some capelin continued to be available to diving seabirds at deeper depths in 2014 and 2015. By 2019, capelin frequencies in diets of surface feeders were near the mean value but remained below the mean in diver diets.

Frequencies of sand lance were below the long‐term mean for diving and surface feeding seabirds during 2008–2013 when water temperatures were cooler, and in 2014–2015 during the first two summers of the MHW (Figure 3a). From 2016–2019, sand lance frequencies in diets of divers were closer to the long‐term mean while negative anomalies persisted in diets of surface feeders.

Although herring frequencies comprised a lower proportion of diets overall (mean frequency over time 0.10 and 0.13 for divers and surface feeders, respectively) compared to sand lance and capelin, they increased in surface feeder diets in 2015 and remained higher than average through 2019 (Figure 3a). Herring frequency anomalies were not consistent between surface feeder and diver diets for more than a decade prior to the MHW but surface feeders in particular relied on herring to a greater extent during and after the MHW, which is consistent with inshore feeding and greater foraging distances by kittiwakes during the MHW (Osborne et al., 2020).

When these time series of forage fish indices from seabird diets were summarized by the portfolio effects (1‐variance ratios), a rapid decline at the onset of the MHW identified strong trophic instability and synchronous collapse of the forage community in the northern GOA in 2014–2015 (Figure 3b). More stable, asynchronous dynamics across forage fish species persisted for at least two decades prior to the MHW, and returned shortly after the MHW dissipated. Similar patterns in portfolio effects between surface feeding and diving seabirds suggest a common underlying mechanism, although synchrony among species in diver diets was more pronounced than it was for surface feeders.

### 3.3 Forage fish trends in survey indices

**FIGURE 4:**
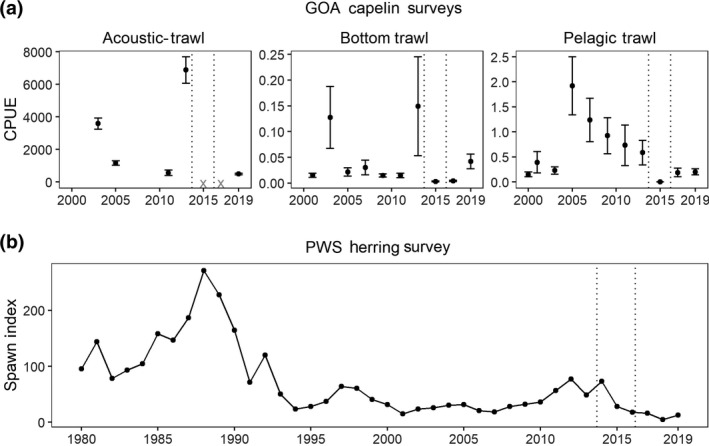
Survey indices for (a) Gulf of Alaska (GOA) capelin (mean ± SE catch per unit effort, CPUE, kg km^−2^) by survey type. Years in which capelin densities were too low to assess during acoustic‐trawl surveys are denoted by ‘*x*’. (b) Prince William Sound (PWS) herring spring spawning index (mile‐days milt). Dotted vertical lines demarcate the timing of the Pacific marine heatwave

Survey‐based indices of relative abundance from the GOA shelf indicate that capelin abundance declined by at least 98% from 2013 to 2015, reaching the lowest values observed in the dataset (2000–2019; Figure 4a). Prior to the MHW, in 2013 all surveys indicated that capelin were either at average or relatively high abundance levels relative to the 20‐year mean. The sharp decline of capelin densities in 2015 was the greatest 2‐year change between 2000 and 2019 in both the bottom and pelagic trawl surveys. Capelin densities were too low during acoustic‐trawl surveys in 2015 and 2017 to be acoustically discriminated from other fish, which prevented estimation of their abundance. Low abundances during 2015 persisted though 2017, indicating that the population had effectively collapsed both across the GOA shelf and within its core area around Kodiak. All surveys observed signs of at least partial recovery during 2019, with the bottom trawl survey indicating that capelin had returned to average abundance levels while the other surveys indicated the population was increasing but remained below average levels. Capelin are patchy in their horizontal and vertical distribution, and the lack of concordance among surveys in some years reflects differences among spatial domains and gear types used in the surveys (McGowan et al., 2020).

Prince William Sound herring spawning activity was at historically low levels during the MHW (Figure 4b). Between springs of 2014 and 2015, the observed spawning activity index decreased by 73%, and continued to decline to lowest recorded levels in 2018. While this population has not recovered since it collapsed in 1993, the recent decline during the MHW followed a modest increase in spawning between 2011 and 2014 that represented the first notable increase in spawning since the late‐1990s.

### 3.4 Forage fish age, length, energy content, and growth

**FIGURE 5:**
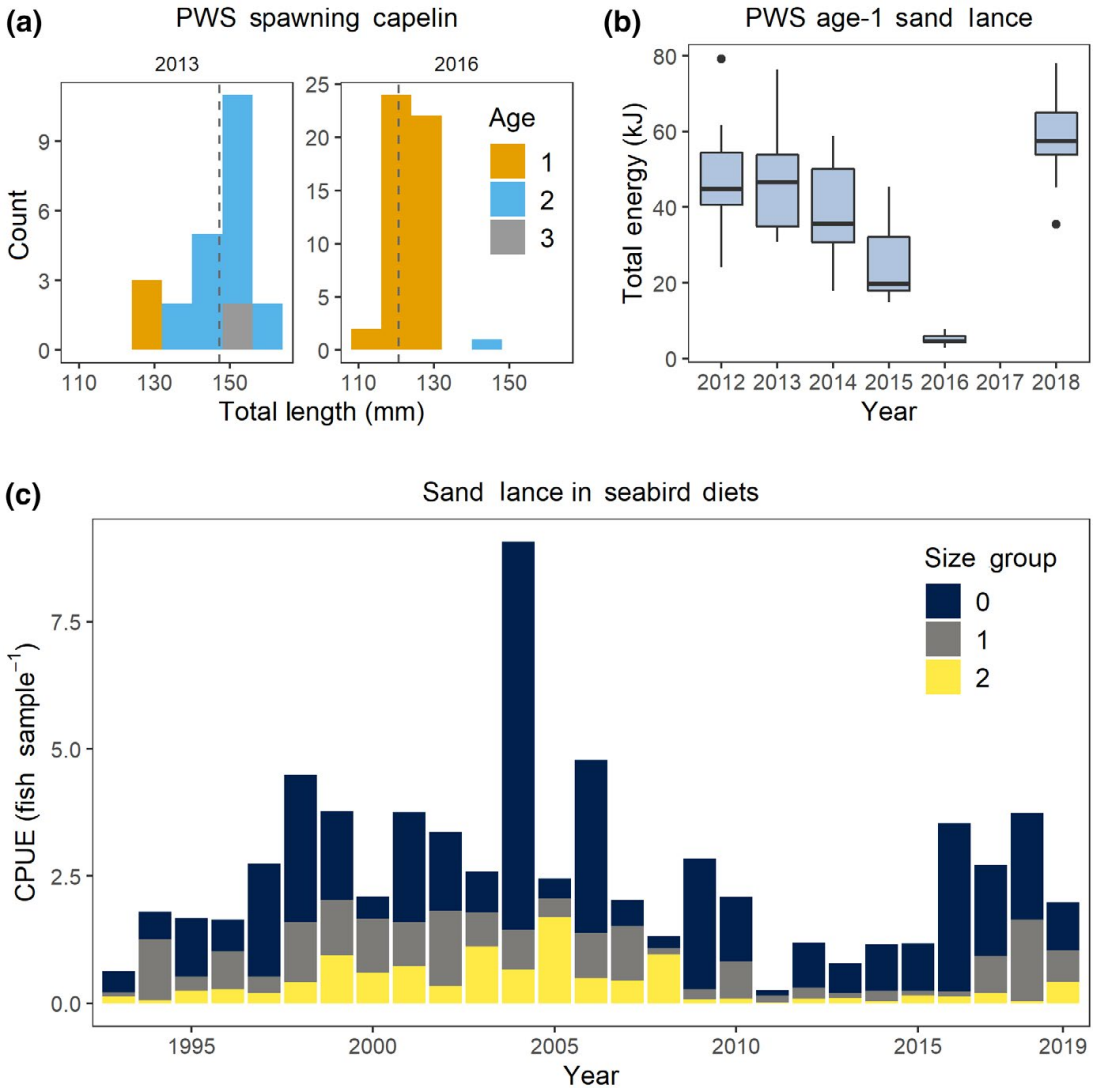
Forage fish age, length, and energy content in the Northern Gulf of Alaska. (a) Length at age histogram from spawning male capelin within Prince William Sound (PWS) during July 2013 and 2016. Dashed line indicates the mean length in each year. (b) Interannual variability of whole fish energy for age‐1 sand lance within PWS. Points represent the values of outliers. (c) Sand lance catch per unit effort (CPUE) by year and size group in seabird diets at Middleton Island during June–August

Spawning capelin in PWS were younger and smaller during the MHW in 2016 than prior to the MHW in 2013 (Figure 5a). Distributions by age shifted from primarily age‐2 fish during 2013 to primarily age‐1 fish during 2016. This shift in age structure suggests recruitment failure by the 2014 year class, and favorable conditions for growth of the 2015 year class to reach length at maturity at age‐1. Still, the fast‐growing age‐1 spawning capelin in 2016 were smaller than spawning capelin in 2013 (124 ± 27 mm vs. 147 ± 19 mm; *t* = 12.391, *p* < 0.001).

Within PWS, the total energy of age‐1 sand lance declined significantly for 2015–2016 compared to 2012–2014 (von Biela et al., 2019). No sampling occurred during 2017, however, by 2018 total energy of age‐1 sand lance had returned to levels that were not significantly different from those observed during 2012 and 2013 (ANOVA; *F*_1,8_  = 146.7; *R* ^2^ = 0.94, *p* < 0.001; Tukey HSD, *α* < 0.05; Figure 5b).

The proportion of larger size classes of sand lance (typically corresponding to >age‐0, von Biela et al., 2019) in seabird diets declined steeply after 2008 and remained low through 2019 (Figure 5c). Low frequency of occurrence and high proportion of the smallest size class (typically corresponding to age‐0) sand lance persisted through the MHW years; however, this pattern began well before the MHW. In 2016, 90% of sand lance sampled by diving seabirds (*n* = 1160) were fish from the smallest size class. Although sand lance availability was approaching the long‐term mean by 2016 (Figure 3a), these fish were mainly small and energetically poor (Figure 5c; von Biela et al., 2019).

**FIGURE 6:**
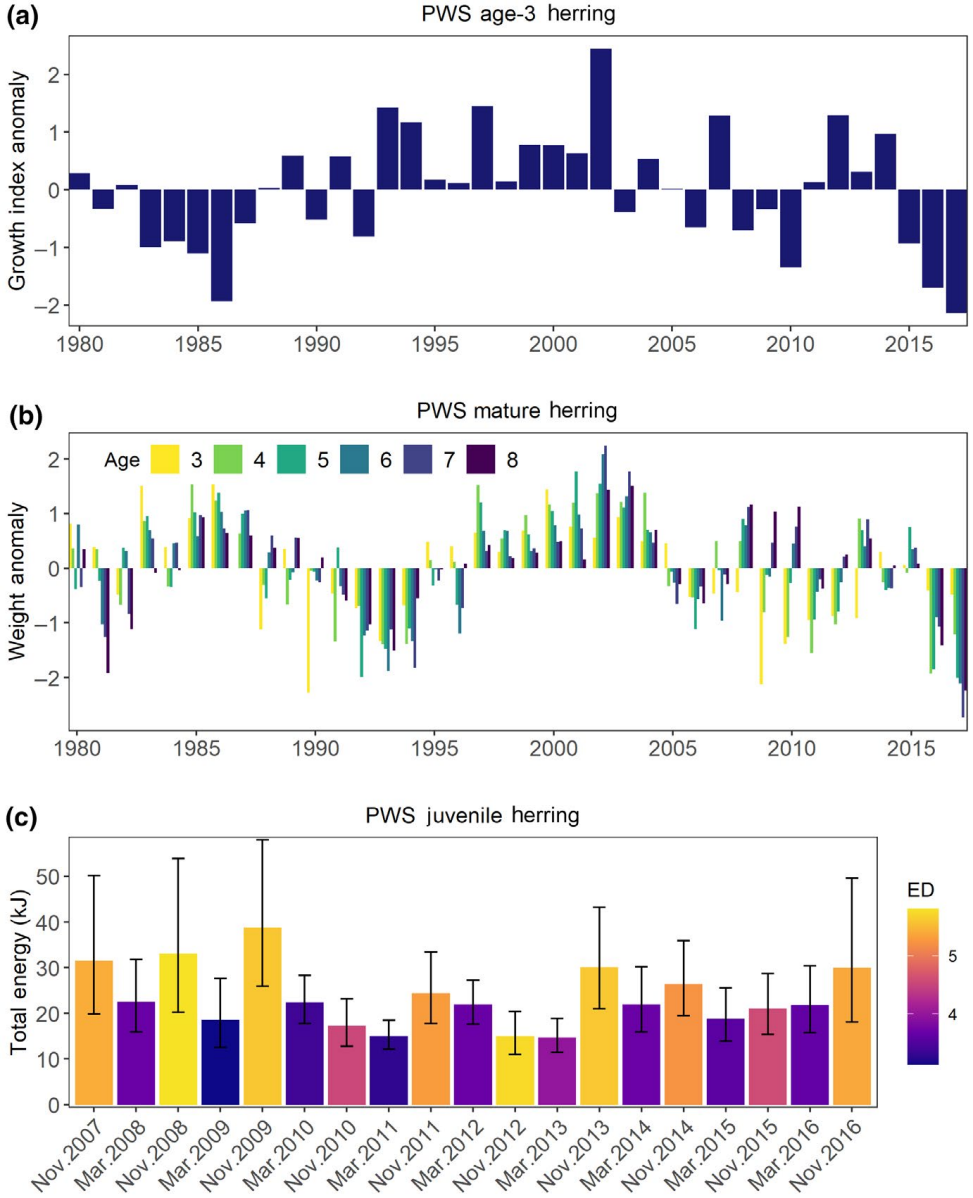
Prince William Sound (PWS) herring indices, (a) age‐3 growth anomalies from scale increment width measurements, (b) spring pre‐spawning weight anomalies, and (c) estimated least square mean (±95% CI) juvenile total energy and mean energy density (ED, kJ g^−1^ wet weight, color) in March and November. Anomalies were standardized to mean 0, SD 1.

Prince William Sound herring growth index anomalies showed a steady decline beginning during 2015 with the lowest values observed during 2017 (Figure 6a). Although the growth anomalies in recent years are not unprecedented in the time series, low growth during 2017 indicates poor growth conditions persisted within the region after the MHW. In contrast, negative growth anomalies in 1986 were of similar magnitude but occurred when the spawning population was reaching its peak (Figure 4b) and may have been related more to density‐dependent factors. Strong negative anomalies in weight‐at‐age of mature herring in spring were observed in 2016 and reached record low values during 2017 for older fish (Figure 6b).

Changes in juvenile herring total energy generally reflect indices of size and energy gain during the growth season between spring hatch and late‐fall in November, and overwinter condition in March. Models to estimate total energy for each month while accounting for random differences across sample locations (as described by Gorman et al., 2018) explained 73% and 50% of the variation in the data from November and March, respectively. Although age‐0 herring total energy was below the mean in November 2015, it was not outside the range of variability seen in previous years based on overlapping confidence intervals (Figure 6c). Furthermore, in stark contrast to sand lance (which were measured in July, not March), the 2015 year class of herring had relatively high total energy and energy density in March 2016 (Figure 6c). The combination of high total energy and high energy density suggests the 2016 year class of herring had relatively high lipid reserves at a large size compared to other years.

### 3.5 Zooplankton indices

**FIGURE 7:**
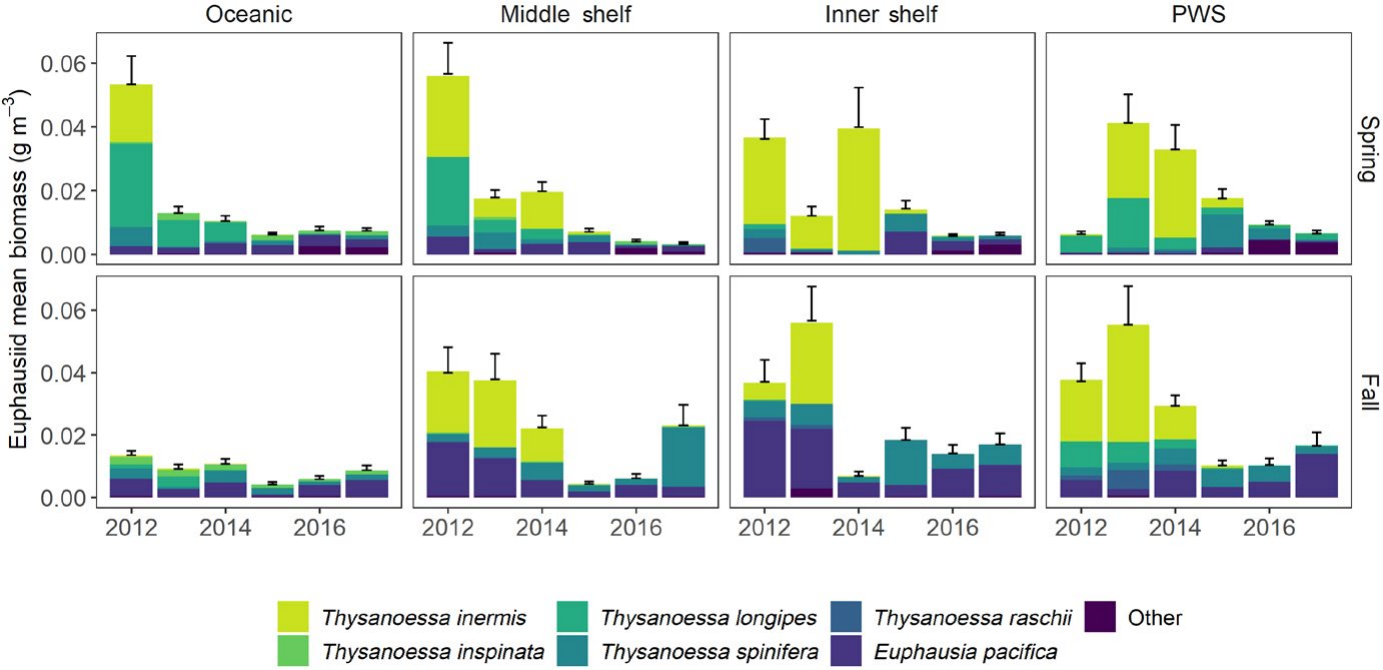
Mean (SD) biomass of euphausiids by year, region, season, and species (color) sampled along the Gulf of Alaska shelf and Prince William Sound (PWS)

In general, euphausiid biomass along the GOA shelf and in PWS was dominated by *Thyanoessa inermis* and *T. longipes* from 2012 to 2014 and mean biomass of these species was reduced abruptly by 2015 (Figure 7). Changes in mean biomass of euphausiids, therefore, were driven by the loss of these species, particularly during spring.

**FIGURE 8:**
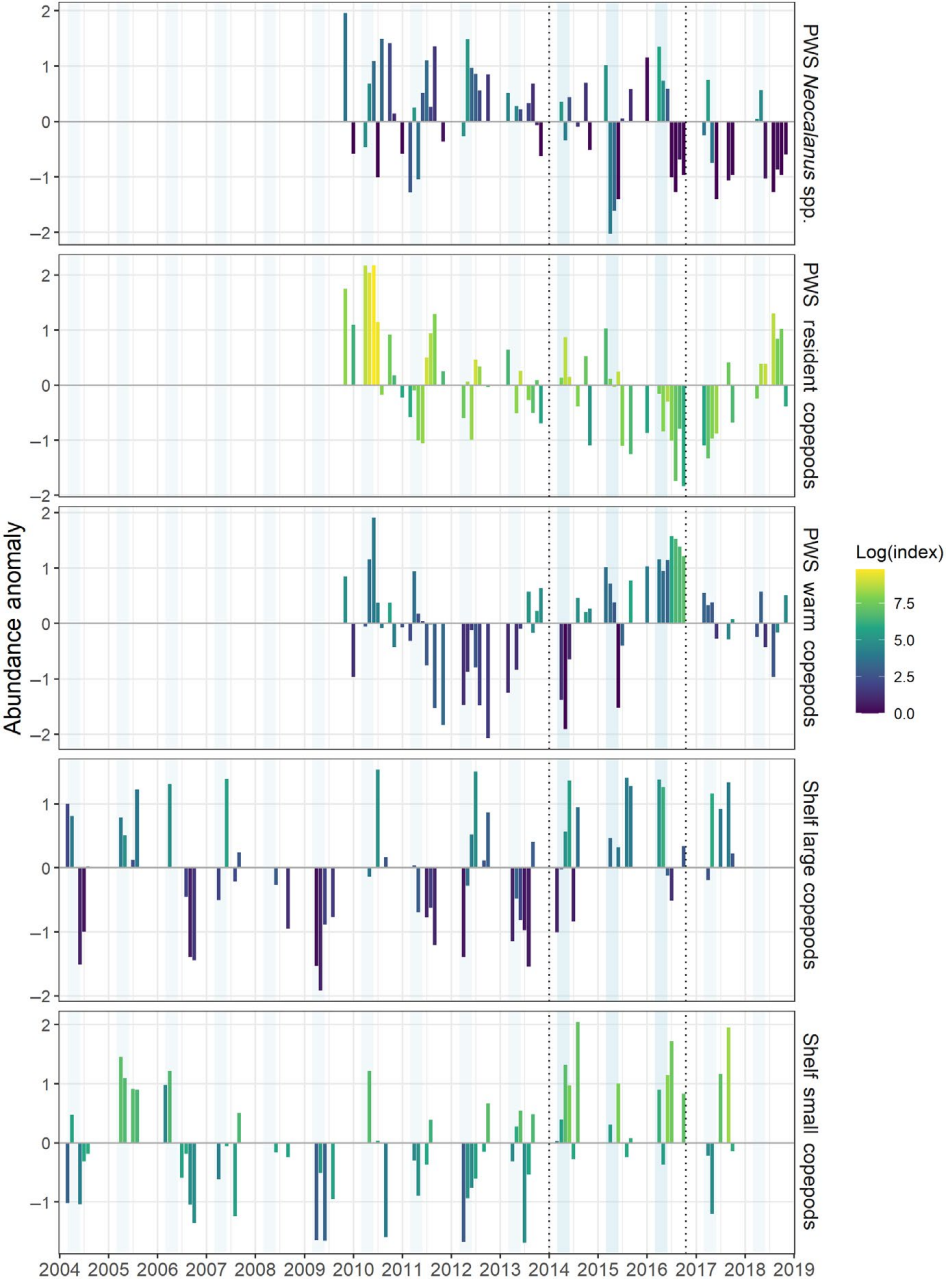
Monthly log‐transformed copepod abundance indices (color) standardized anomalies (bars) for species groups sampled in Prince William Sound (PWS, bongo net, m^−3^) and along the Gulf of Alaska shelf (continuous plankton recorder, sample^−1^). Dotted horizontal lines demarcate the timing of the Pacific marine heatwave, and spring months (March–May) are shaded in light blue

Within the bays of PWS abundance anomalies of the dominant large copepod *Neocalanus* spp. were as much as 2 SD below the mean in spring 2015, and >1 SD above the mean in spring 2016. Resident copepods and warm water copepods had inverse trends, which signaled a sustained community shift due to the influx of warm water copepods that occurred especially in summer and fall 2016 (Figure 8). In contrast to PWS, copepod data from shelf regions in the northern GOA indicated that abundance anomalies were generally positive for both large and small copepods during the MHW.

**FIGURE 9:**
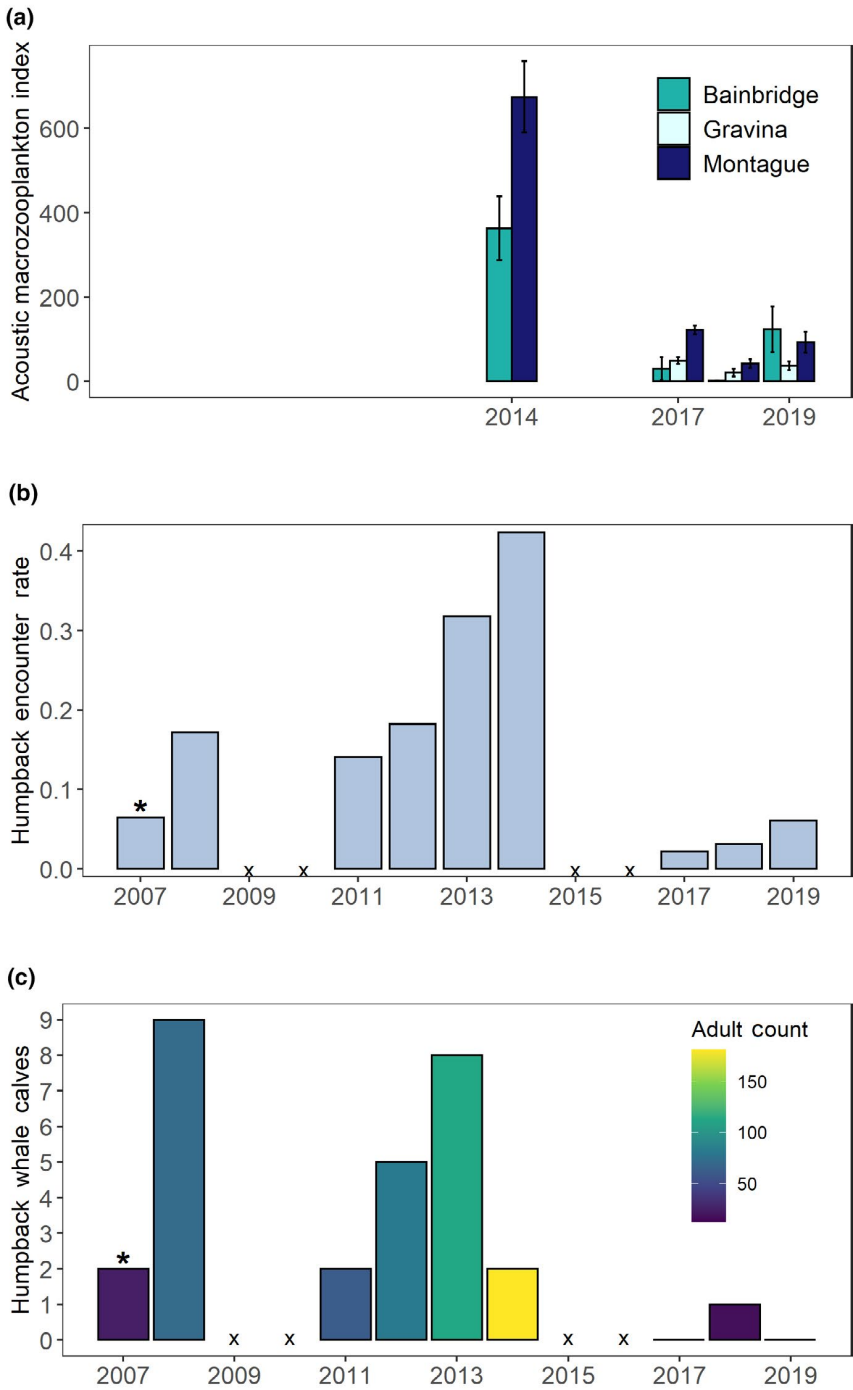
Prince William Sound humpback whale indices. (a) Acoustic macrozooplankton index (nautical area scattering coefficient, m^2^ nmi^−2^) sampled within humpback whale foraging locations during fall. (b) Humpback whale encounter rates (individuals km^−1^). *Note the survey in 2007 was incomplete. (c) Number of calves and adult counts (color) observed on surveys

The acoustic index for PWS macrozooplankton in fall suggests that densities declined in key regions with foraging predator aggregations after 2014 (Figure 9a; Figures S1 and S2).

### 3.6 Marine predators

Marine predators specializing on pelagic forage species in the northern GOA experienced shifts in distribution, reduced encounter rates, and reduced reproduction. PWS humpback whale encounter rates increased on fall surveys through 2014 and declined by 2017 when surveys were next conducted (Figure 9b). The number of calves observed on surveys decreased after 2013, however, even while the abundance of adults in PWS peaked the next year. By fall 2019, humpback whale encounter rates increased slightly but had not returned to pre‐MHW levels. Between fall 2014 and fall 2017, acoustic macrozooplankton index in humpback foraging habitat declined (Figure 9a). Reduced macrozooplankton biomass, along with historically low herring indices in PWS (Figure 4b), may largely explain the reduction in humpback whale encounter rates in the region.

**FIGURE 10:**
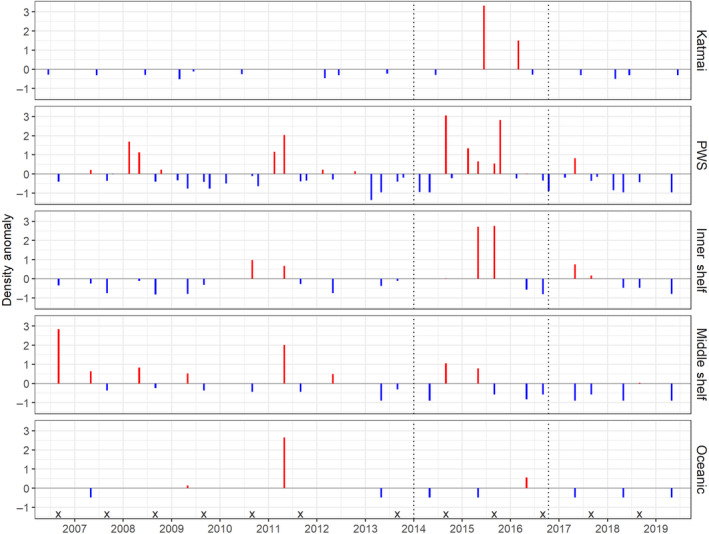
Murre (*Uria* spp.) standardized density anomalies (mean of 0 and SD of 1 for each season and region). September surveys in the oceanic region, in which no murres were observed, are indicated by ‘*x*’. Dotted horizontal lines demarcate the timing of the Pacific marine heatwave. PWS, Prince William Sound

Unusually high densities (>2 SD) of murres (Figure 10) were observed within PWS waters during fall 2014 and late‐fall 2015, within nearshore areas of Katmai during summer 2015, as well as for the inner shelf during spring and fall 2015 (Figure 10). These high densities suggest inshore movement of murres preceded mass mortality of the species during winter 2015–2016 (Piatt et al., 2020).

## 4 DISCUSSION

A primary mechanism of trophic disruption in the multi‐year MHW was that it reduced the abundance of several key forage fishes and some cold‐water‐associated euphausiids (*T. inermis* and *T. longipes*) while it simultaneously lowered nutritional quality of many forage species. A steep, but short‐lived decline in portfolio effects at the onset of the MHW points to strong trophic instability in the system due to synchronous collapse of the forage fish community. Diversity of life‐history strategies, including differential tolerances to water temperature and seasonality of spawn timing or growth phases, typically promotes staggered population trajectories of forage species; however, this mechanism for creating stability in marine food webs apparently failed during the MHW.

The MHW was in fact a relatively brief heating event compared to the 1977 North Pacific Regime Shift, which was driven by the Pacific Decadal Oscillation and involved a similar temperature range in its transition from cool to warm anomalies but played out over more than a decade rather than just 2 years (Anderson & Piatt, 1999; Hare & Mantua, 2000; Litzow & Mueter, 2014). This climate oscillation led to a complete reorganization of pelagic food webs in the GOA and restructured marine communities, from phytoplankton to whales (Chavez et al., 2003; Francis et al., 1998). Despite the magnitude of biological change wrought by regime shifts, which by definition imply large‐scale reorganizations of marine ecosystems (Overland et al., 2008), those changes are not generally evidenced in abrupt and short‐term impacts such as wholesale recruitment failures, precipitous population declines, or mass die‐off events (Chavez et al., 2003; Litzow & Mueter, 2014; Yatsu et al., 2008).

So, despite its relatively brief tenure in the NE Pacific, the MHW elicited an abrupt response among many members of the pelagic food web. In this respect, “heatwaves” appear qualitatively different from “regime shifts” (Litzow et al., 2020), and this begs for a mechanistic explanation of how and why heatwaves disrupt food webs.

### 4.1 Simultaneous collapse of the middle trophic level

When we examined each of the three GOA forage fish species in detail, we found differing life‐history traits that promote staggered year class strength, and accordingly, different population trends among species. For example, following an extended period of cold water conditions in the northern GOA just prior to 2014, capelin declined abruptly at the onset of the MHW in 2014, whereas herring declined a year later. Sand lance generally followed a different trend, being relatively uncommon during several cold years prior to the MHW (Hatch, 2013), but failed to increase at the onset of warmer MHW conditions as would have been anticipated from patterns observed in the previous decades (Speckman et al., 2005; Sydeman et al., 2017). Other work has shown that capelin also disappeared abruptly in diets of Pacific cod between 2013 and 2015 (Barbeaux et al., 2020), supporting our findings from seabird diets and region‐wide acoustic and trawl survey data (McGowan et al., 2020, this study). To our knowledge, the only other forage fish collapse of a similar scale on record occurred in the Barents Sea in 1986–1987, when a common murre die‐off also coincided with simultaneously low stock biomass of capelin (*Mallotus villosus*), Atlantic herring (*Clupea harengus*), and juvenile Atlantic cod (*Gadus morhua*; Erikstad et al., 2013).

Species‐specific thermal optima are not well known, but because capelin is a more northern species associated with cooler ocean temperatures (Sydeman et al., 2017; Thompson et al., 2019) it is likely that under warming conditions their distribution contracted or shifted locally to deeper, cooler waters (Arimitsu et al., 2008; McGowan et al., 2019; Rose, 2005). In contrast, sand lance and herring have broader distributions, and may be more tolerant of warmer temperatures (Speckman et al., 2005). For example, sand lance were positively associated with warmer conditions in the GOA during several decades prior to the MHW (Abookire & Piatt, 2005; Sydeman et al., 2017; Thompson et al., 2019), but demonstrated a strong negative response in growth and whole‐body energy content in PWS during the MHW (von Biela et al., 2019). Similarly, age‐0 herring growth was positively related to higher diatom abundance and warmer temperatures in PWS, although the relationship broke down under extreme temperatures (Batten et al., 2016). The negative response in growth and energy content of sand lance during the MHW suggests the temperature optima may have been exceeded, which has been demonstrated in other species (Laurel et al., 2016).

### 4.2 Truncation of age and size structure in forage fish

Truncation of the older, larger size classes in capelin and sand lance, both species with relatively short life spans, could have been due to top‐down pressures during the MHW. Changes in the demographic structure of fishes are a well‐documented consequence of size‐selective removal processes such as exploitation due to predation, disease, or fishing (Ohlberger et al., 2014; Perry et al., 2010). Such size truncation reduces the buffering capacity of the population to environmental variability leading to poor recruitment and survival (Perry et al., 2010; Planque et al., 2010). It can also alter spawning dynamics. For example, smaller, younger individuals produce fewer eggs, since fecundity is strongly related to size (Gjøsæter, 1998), and spawning by younger populations may occur over a reduced duration and spatial extent (Perry et al., 2010).

In the absence of forage fish fisheries in the region, top‐down pressures on forage species during the MHW are hypothesized to have primarily resulted from increased metabolic needs of large predatory fish over multiple years of warm conditions (Barbeaux et al., 2020; Piatt et al., 2020). A 2°C increase in temperature would have increased the daily rations of GOA pollock, Pacific cod, and arrowtooth flounder (*Astheresthes stomias*) by 70%, 34%, and 65%, respectively (Holsman & Aydin, 2015). Moreover, owing to a strong year class in 2012, the walleye pollock biomass in the region nearly doubled in the GOA between 2013 and 2015 (Dorn et al., 2019), which may also have led to a rapid increase in predation pressure on prey species. The sustained warm temperatures spanning two winters, along with increased consumption needs by ectothermic predatory fish, could have provided a mechanism for large‐scale top‐down control of the middle trophic level.

Disease has also been identified as a top‐down stressor limiting the PWS herring population. The primary pathogens are Viral Hemorrhagic Septicemia virus, with infection rates increasing when schools occur in dense aggregations such as during periods of spawning or predator‐corralling, and Ichthyophonus, which tends to be more prevalent with increasing host size and age (Hershberger et al., 2010, 2016). No relationship between warming temperatures and disease prevalence has been established, but disease is considered an underlying stressor that may be a factor in the failure of the PWS herring population recovery.

### 4.3 Decreased quality of forage fish

Lower quality of forage fish, manifested through lower growth and/or energy content, are suggestive of bottom up pressures including poor quality zooplankton prey in combination with thermal stress. In 2016, age‐1 sand lance in PWS were 89% lower in total energy content than in previous years when cooler temperatures prevailed (von Biela et al., 2019). Similarly, pre‐spawning herring showed signs of lower body condition, including lower growth and weight‐at‐age (this study). A zooplankton community shift to one dominated by (less nutritious) small copepods may be related to negative growth anomalies and poor condition of forage fish if the prey supply was insufficient to meet increased consumption demands of planktivorous predators in warmer temperatures. On the other hand, total energy of the 2015 herring year class was not out of the range of normal variability in fall 2015 or spring 2016. The contrast between sand lance and herring in this regard may be related to behavioral differences, as sand lance are known to estivate under conditions of extreme temperatures (Tomiyama & Yanagibashi, 2004), whereas juvenile herring remain in the water column to feed, particularly in warm temperatures when metabolic rates are higher (Sewall et al., 2019).

### 4.4 Shifts in zooplankton communities

Euphausiid biomass and macrozooplankton abundance indices decreased in the region after 2014 (this study). Lower euphausiid biomass was driven by the loss of *T. inermis* and *T. longipes*, both species that are associated with cooler temperatures (Iguchi & Ikeda, 2005; Pinchuk et al., 2008). Pinchuk et al. (2008) found *T. inermis* abundance was positively associated with a cool regime in 1999–2002, perhaps because the species has a relatively high mass‐specific metabolic rate thus spends significantly more energy on metabolism during warmer winters. Following a warm winter, *T. inermis* would have less energy reserves in spring for spawning.

While euphausiids declined in biomass during the MHW, copepod biomass generally increased on the shelf, which is likely related to faster copepod development rates in warmer water (Kimmel & Duffy‐Anderson, 2020). Although warmer temperatures would also have shortened generation times of the smaller taxa that dominated in summer, increased abundances were driven largely by contributions from smaller genera within both size classes that would have effectively lowered mean body size of the community (Batten et al., 2018).

Patterns of copepod abundance in PWS were more complex during the MHW. For example, strong negative anomalies in abundance of the dominant large‐bodied copepods *Neocalanus* spp. during spring 2015 were followed by positive anomalies in spring 2016. Meanwhile, inverse trends in resident vs. warm water signaled a community shift (McKinstry & Campbell, 2018). These lower trophic data suggest that food availability and quality may have contributed to observed changes in planktivorous forage fish populations during this time.

### 4.5 Impacts to pelagic predators

The magnitude of this system‐wide perturbation was evidenced in a coherent response of several dominant top predators within each of the important marine vertebrate classes. In birds, for example, there was a mass‐starvation die‐off of an estimated 0.5–1.2 million common murres in the NE Pacific during 2015–2016, widespread total reproductive colony failures in 2015–2018, and mass mortalities of several other seabird species (Jones et al., 2018; Piatt et al., 2020). Although the murre die‐off occurred along the entire US west coast, densities were greatest in PWS and coastal regions along the GOA during winter 2015–2016 (Piatt et al., 2020) following a marked redistribution of the species to inside and coastal waters (this study). In mammals, there was mass mortality of humpback and fin whales in the GOA during 2015–2016 (Savage, 2017), as well as precipitous declines in abundance and calf production rates of humpback whales throughout the region (this study, Neilson & Gabriele, 2019). Similarly, in fish, Barbeaux et al. (2017) showed there was a large (>71%) reduction in biomass of Pacific cod from 2015 to 2017 resulting in a severe (77%) reduction in cod fishing quotas. Moreover, increased mortality and diminished body condition of Pacific cod during the MHW were attributed to increased metabolic demands and nutritional stress during the protracted warm conditions (Barbeaux et al., 2020).

### 4.6 Implications

The large spatial scale in which mass starvation of large‐bodied pelagic specialist predators was associated with the MHW suggest our aggregate findings, despite the disparate spatial and temporal domains within the datasets compiled here, are representative of the larger GOA marine ecosystem. This information provides important context in understanding the response of large marine ecosystems to current and future events. For example, similar to our findings, recent severe heatwave conditions across both hemispheres have been associated with zooplankton community shifts, species range expansions, and unusual mortality in pelagic predators (Evans et al., 2020; Romano et al., 2020; Ruthrof et al., 2018).

Marine heatwaves such as this are associated with climate change and are increasing in frequency and duration globally (Laufkötter et al., 2020; Oliver et al., 2018). The 2014–2016 MHW caused a large‐scale disruption of the pelagic food web, manifested through a simultaneous reduction in the abundance and quality of key forage taxa. The MHW appeared to overwhelm a generally diverse and resilient forage community, leading to catastrophic failures in top pelagic predators and eliminating any ecological advantages that might have accrued from having a suite of coexisting forage species with differing life‐history compensations (“portfolio effect”).

Changes at lower and upper trophic levels provide evidence for a large‐scale reduction in energy transfer through the middle trophic level (forage fish and euphausiids) during the MHW. Declines in the abundance and quality of forage fish and euphausiids were likely a primary driver of the mass mortalities, changes in distribution, reproductive failure, and malnutrition observed in piscivorous marine predators that depend on the pelagic food chain.

The ecosystem response observed appears fundamentally different from temperature‐induced “regime shifts,” which transition between cold and warm phases without accompanying disruptions in energy flow, as evidenced by large‐scale mortality of seabirds, whales, and predatory fish. The abrupt but brief decline in portfolio effects by the forage fish community during this event appears to differentiate ecosystem responses to “regime shift” versus “heatwave” temperature anomalies and warrants further investigation for understanding and anticipating the response of large marine ecosystems to future heatwave events.

**TABLE 1:** Description of datasets used to identify changes in availability, abundance, and quality of forage species, as well as coincident changes in lower and upper trophic‐level taxa in the Northern Gulf of Alaska (GOA) during the 2014–2016 Pacific marine heatwave.

Purpose	Taxa	Region, Index (units)	Years, Source
Middle trophic level: Forage fish abundance indices PWS	Forage Fish	GOA summer frequency in seabird diets	1993–2019, GWA
	Capelin	GOA summer acoustic‐trawl, pelagic trawl, and bottom trawl survey anomalies (CPUE, kg km^−2^)	2000–2019, NOAA AFSC
	Herring	PWS spring aerial spawning index (mile‐days milt)	1980–2019; ADF&G
Middle trophic level: Forage fish quality indices	Capelin	PWS July male spawning age (years), length (total length, mm)	2013, 2016; GWA
	Sand lance	GOA summer size proportional CPUE in seabird diets (fish sample^−1^)	1993–2019, GWA
	Sand lance	PWS July age‐1 total energy (kJ)	2012–2016, 2018; GWA
	Herring	PWS age‐3 annual growth increment anomaly (mm), and weight at age anomaly (g)	1979–2017; ADF&G
	Herring	PWS March, November age‐0 whole fish total energy (kJ)	2007–2016; HRM
Lower trophic level: Zooplankton abundance indices	Euphausiid	GOA shelf/PWS May, September mean biomass (g m^−3^)	2012–2017; GWA
	Copepod	PWS monthly mean abundance by species group (m^−3^)	2012–2017, GWA
	Copepod	GOA shelf monthly mean abundance by species group (sample^−1^)	2009–2018, GWA
	Macrozooplankton	PWS September acoustic macrozooplankton index (nautical area scattering coefficient, m^2^ nm^−2^)	2014, 2017–2019; GWA
Upper trophic level: Marine predator indices	Humpback whale	PWS September encounter rate (individuals effort^−1^)	2008–2015, 2017–2019; GWA
	Humpback whale	PWS September crude index of birth rate (calves individual^−1^)	2008–2015, 2017–2019; GWA
	Murre	GOA/PWS/Katmai density anomaly (birds km^−2^)	2006–2019; GWA

Abbreviations: ADF&G, Alaska Department of Fish and Game; CPUE, catch per unit effort; GWA, Gulf Watch Alaska long‐term ecosystem monitoring program; HRM, Herring Research and Monitoring Program; NOAA AFSC, National Oceanic and Atmospheric Administration Alaska Fisheries Science Center; PWS, Prince William Sound.

## Supporting information

Fig S1‐S2

## Data Availability

The data that support the findings of this study are openly available in repositories as cited, for example https://doi.org/10.5066/F74J0C9Z, https://doi.org/10.5066/P96N5PVE, and references to data publicly available at: https://portal.aoos.org/gulf‐of‐alaska.
