# Supplementary material for: Heatwave‐induced synchrony within forage fish portfolio disrupts energy flow to top pelagic predators
Source: Glob Chang Biol. 2021 Mar 6;27(9):1859–78. doi: 10.1111/gcb.15556 (PMC8048560; doi:10.1111/gcb.15556)
Supplement: Supplementary file 1 — Fig S1‐S2 [file GCB-27-1859-s001.docx]

Supplementary Information


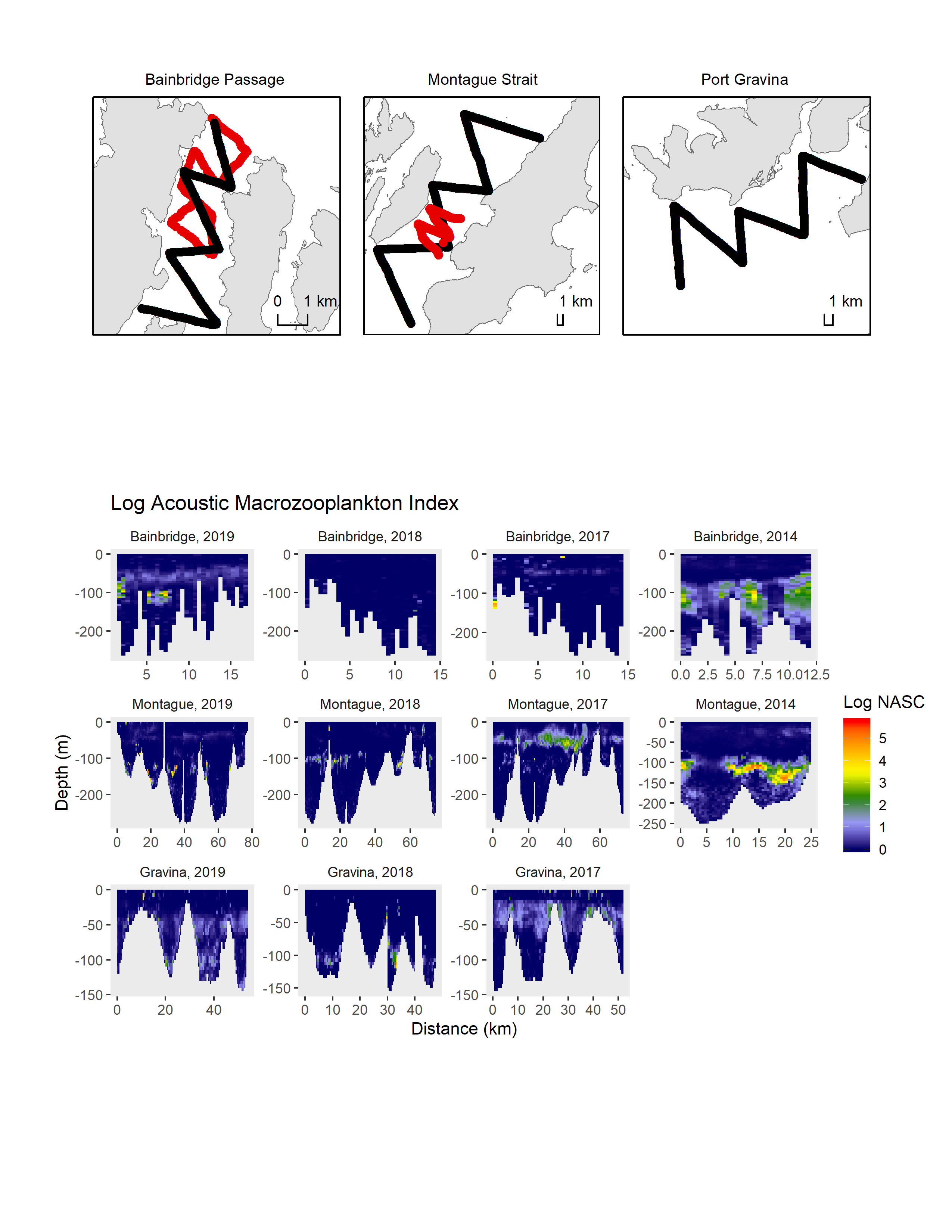


Figure S1. Maps of acoustic transects (black lines) from three humpback whale foraging aggregation regions within Prince William Sound, Alaska. The transects conducted in 2017-2019 were based on the pilot work conducted in 2014 (red lines).

Figure S2. Acoustic index of macrozooplankton relative to water column depth (y-axis) and distance along transects (x-axis, note differing scale for each plot based on effort). Each 5 m depth by 0.5 km grid cell represents the log-transformed mean NASC due to organisms with scattering properties of macrozooplankton. Note that effort in Montague Strait in 2014 covered only a fraction of the survey area in 2017 and 2018 (see Fig. S1). Below bottom depth cells are represented in grey.
